# An Mpox Multi-Antigen-Tandem Bivalent mRNA Candidate Vaccine Effectively Protects Mice Against the Vaccinia Virus

**DOI:** 10.3390/vaccines13040374

**Published:** 2025-03-31

**Authors:** Jun Zuo, Jiayu Wu, Zhen Zhang, Jinrong Long, Changxiao Yu, Yuqin Liao, Hongsheng Zhang, Jing Yang

**Affiliations:** 1 College of Chemistry and Life Science, Beijing University of Technology, Pingleyuan 100#, District of Chaoyang, Beijing 100124, China; 2 Bioinformatics Center of AMMS, Beijing 100850, China

**Keywords:** mpox, tandem, bivalent, mRNA vaccine, VACV

## Abstract

Background: Since the outbreak of mpox in 2022, the disease has spread rapidly worldwide and garnered significant public attention. Vaccination is regarded as an effective measure to prevent the spread of mpox. The success of the COVID-19 mRNA vaccine demonstrates that mRNA-based vaccines represent a rapid and multifunctional platform with considerable potential, and are expected to be a strategy to address mpox spread. Methods: In this study, we screened an mpox multi-antigen-tandem bivalent mRNA vaccine candidate: a lipid nanoparticle-encapsulated mRNA-1017 and mRNA-1995 (mRNA-3012-LNP). We then evaluated the immunogenicity of the mpox virus (MPXV) bivalent mRNA vaccine candidate and its protective efficacy against the vaccinia virus (VACV) in a mouse model. Results: Mice vaccinated with two doses of the mRNA-3012-LNP vaccine exhibited robust binding antibody responses and MPXV-specific Th-1-biased cellular immune responses in vivo. Notably, the boosted immunized mice generated potent neutralizing antibodies against the VACV, effectively protecting them from viral challenge. Additionally, serum transfer protection experiments indicated that serum from mice inoculated with mRNA-3012-LNP was effective in protecting nude mice from VACV challenge. Conclusions: Our results suggest that the mpox bivalent mRNA candidate vaccine mRNA-3012-LNP induces strong immunogenicity and has the potential to serve as a safe and effective vaccine candidate against mpox epidemics.

## 1. Introduction

Mpox is a zoonotic viral disease caused by the mpox virus (MPXV). Mpox was first isolated from monkeys in 1958, and the first human case of MPXV infection was reported in the Democratic Republic of Congo (DRC) in 1970 [1]. For an extended period, mpox primarily spread in West and Central African countries until May 2022, when a sudden outbreak of mpox occurred and rapidly spread worldwide. From July 2022 to May 2023, the World Health Organization classified the mpox outbreak as a public health emergency of international concern (PHEIC) [2,3,4]. However, in 2023, the DRC experienced another significant mpox outbreak that quickly disseminated throughout Africa [5]. From 14 August 2024 to the present, mpox has once again been classified as a PHEIC [6]. As of 9 March 2025, approximately 129,000 confirmed cases and around 290 deaths have been reported globally [7]. These situations demonstrate the dangers of the mpox virus, and call for the development of appropriate strategies.

Mpox virus (MPXV) belongs to the *Orthopoxvirus* (*OPXV*) genus in the *Poxviridae* family [8], which also includes cowpox virus (CPXV), variola virus (VARV), and vaccinia virus (VACV). MPXV has two branches, branch I (including Ia and Ib) and branch II (including IIa and IIb) [9,10]. The multinational mpox outbreak of 2022 was attributed to a branch IIb virus and was characterized by low severity, including symptoms such as fever, headache, swollen lymph nodes, skin lesions, and generalized blisters and pustules, as well as hemorrhagic tendencies [11]. In contrast, the 2024 outbreak of mpox was primarily caused by branch Ib viruses and proved to be significantly more lethal than those of branch IIb [10,12,13]. Orthopoxviruses are enveloped viruses with double-stranded DNA genomes, exhibiting a high degree of antigenic similarity within the genus [14]. Related studies indicate that the immune response elicited by VACV is cross-protective against other viruses in the Orthopoxvirus genus [15,16,17]. An earlier study also demonstrated that individuals vaccinated against smallpox had an 85% protection rate against MPXV infection [18]. Vaccination remains an effective measure to prevent MPXV infection. In light of the current situation, the World Health Organization has approved two vaccines for the emergency prophylaxis of mpox: MVA-BN and LC16m8. MVA-BN is a non-replicating, third-generation live attenuated vaccine derived from the Modified Vaccinia Ankara (MVA) strain [19,20], while LC16m8 is a minimally replicating live attenuated vaccine based on the Lister/Elstree strain of cowpox virus [21,22]. Additionally, OrthpoxVac (VACΔ6) [23] and ACAM2000 [24] were approved for MPXV prophylaxis in Russia and the United States, respectively, in 2022. ACAM2000 is a second-generation live attenuated vaccine based on the replication of VACV, which may lead to serious adverse reactions during vaccination, particularly in immunocompromised patients [25,26]. In comparison, MVA-BN and LC16m8 demonstrate improved safety profiles. However, in 2022, one study showed that MVA-BN vaccination induced low levels of MPXV neutralizing antibodies [27]. Moreover, the limited availability of MVA-BN and LC16m8 fails to meet the global demand for mpox vaccination. OrthpoxVac (VACΔ6) represents a fourth-generation vaccine developed by the Russian State Research Center for Virology and Biotechnology; however, it lacks extensive published clinical data to support its efficacy. Consequently, there is an urgent need to develop a new generation of MPXV vaccines as a potential solution to the mpox epidemic.

The mRNA-based vaccines have demonstrated significant potential in recent years, offering advantages such as high safety, the effective induction of immune responses, and rapid, scalable production [28]. During the COVID-19 pandemic of 2022, BNT162b2, rapidly developed by Pfizer-BioNTech, and mRNA-1273, developed by Moderna, were granted FDA-approved emergency use authorization and exhibited excellent clinical data, indicating that mRNA-based vaccines are effective in preventing infectious diseases [29]. Consequently, the development of mRNA-based vaccines for MPXV represents the most promising strategy for addressing mpox epidemics. Orthopoxviruses exhibit two distinct forms of infection: intracellular mature virion (IMV) and extracellular enveloped virion (EEV), each possessing a unique set of antigenic proteins. In a previous study, the IMV surface proteins (A27L, L1R, and D8L) and EEV surface proteins (A33R and B5R) of VACV were utilized in the design of a DNA vaccine [30,31]. These studies demonstrated that a DNA vaccine encoding five VACV antigenic proteins (A27L, A33R, L1R, B5R, and D8L) offered superior protection for mice compared to a vaccine targeting a single antigen. In 2023, our group prepared two mpox tetravalent mRNA vaccines, mRNA-A-LNP and mRNA-B-LNP [32]. Both vaccines were able to express four antigenic proteins: A29L, A35R, B6R, and M1R, and they successfully induced an effective immune response in mice, providing significant protection against VACV attacks. In 2024, BioNTech and Moderna developed two mpox mRNA vaccines, BNT166a [33] and mRNA-1769 [34,35], respectively. Both vaccines express a variety of MPXV antigenic proteins that induce a robust immune response in immunized animals and provide effective protection for monkeys exposed to lethal doses of MPXV. Furthermore, both vaccines demonstrated excellent cross-protection against other Orthopoxviruses. This indicates that an ideal MPXV mRNA vaccine should comprise a combination of multiple EEV surface protein and IMV surface protein coding sequences. However, utilizing multiple mRNA mixtures would significantly increase the complexity and cost of mRNA vaccine preparation. Therefore, we considered whether it is possible to reduce the number of mRNAs included in an mRNA vaccine based on the expression of multiple MPXV antigenic proteins as a way to reduce the complexity and cost of vaccine preparation while being able to elicit an excellent immune response.

In the current study, we screened an MPXV multi-antigen-tandem bivalent mRNA vaccine candidate: mRNA-3012-LNP, which encodes the expression of multiple MPXV antigenic proteins, including A29L, A35R, M1R, B6R, and E8L. The bivalent mRNA vaccine candidate elicited excellent antibody responses and antigen-specific T-cell immune responses in mice. In addition, the MPXV bivalent mRNA vaccine candidate provided excellent protection against live VACV virus challenge in mice. In conclusion, these data indicate that mRNA-3012-LNP has the potential to be an available vaccine for the prevention of mpox virus infection.

## 2. Materials and Methods

### 2.1. Cells and Viruses

Cells were incubated in a sterile environment at 37 °C with a 5% CO_2_ concentration. HEK-293T, 143TK, and Vero cell lines were grown in Dulbecco’s Modified Eagle’s Medium (DMEM, Gibco, Miami, FL, USA), which was enriched with 10% fetal bovine serum (Thermo Fisher Scientific, Waltham, MA, USA) and a penicillin–streptomycin mixture (Hyclone, Cytiva, Marlborough, MA, USA). The 143TK cell line was utilized for amplifying the rTV-Fluc (engineered vaccinia virus carrying the firefly luciferase reporter gene), which was sourced from the China National Institutes for Food and Drug Control. Vero cells were used to perform the titration of the rTV-Fluc.

### 2.2. Animals

All animals used in this study were obtained from Beijing Vital River Animals Technology Co. (Beijing, China). The BALB/c mice (SPF class) and nude mice (SPF class) were housed in a carefully controlled breeding facility throughout the study. This facility not only provided the mice with an abundant supply of food and drinking water but also maintained a 12 h light and 12 h dark cycle, precisely regulated to align with the biological rhythms of the mice and ensure their healthy growth. All animal experimental procedures in this study, from the design of the experiments to their implementation, were strictly reviewed and approved by the Animal Experimentation Committee of the China AMMS Laboratory Animal Center under approval number IACUC-DWZX-2022-576.

### 2.3. mRNA Design

The mRNA consists of five main parts: the 5′ end cap structure, a 5′-untranslated region (5′-UTR), an open reading frame (translated region), a 3′-untranslated region (3′-UTR), and a poly (A) tail encoding a vaccine antigen [36,37]. We selected the mpox gene sequence with GeneBank ID PP098593.3 for the coding region and used the structure prediction website (https://dtu.biolib.com/DeepTMHMM (accessed on 12 October 2023)) to predict the extracellular domains (ECD) of MPXV antigenic proteins (A29L, A35R, B6R, M1R, and E8L). The different antigenic protein amino acid sequences were then linked using GS linker (a flexible linker composed of glycine and serine). The designed sequences were then codon optimized and finally, the desired mpox mRNA vaccine sequences were obtained.

### 2.4. Preparation and Characterization of MPhXV-mRNA

Plasmids containing the mRNA coding sequence were digested using BsaI-HFv2 (Biolabs, Burlington, VT, USA) to obtain a linearized DNA template for subsequent reactions. In vitro transcription reactions were performed using the T7 High Yield RNA Transcription Kit (Vazyme, Nanjing, China). The Cap 1 structure was added to the transcription product using the Vaccinia Capping System (Vazyme) and mRNA Cap 2′-O-Methyltransferase (Vazyme). The capping reaction products were thoroughly mixed with a 5M ammonium acetate solution, then placed on ice for 15 min to allow the complete precipitation of the mRNA. The mixture was centrifuged at 12,000 rpm for 15 min at 4 °C. After centrifugation, the supernatant was removed, and 70% iced ethanol was added to gently rinse the mRNA precipitate. Three hours later, the iced ethanol was completely removed, and enzyme-free sterile water was added to the mixture. After the mRNA had fully solubilized, it was stored at −80 °C. The mRNA was subsequently analyzed using an Agilent 2100 Bioanalyzer (Andover, MA, USA).

### 2.5. Validation of mRNA Expression

Each mRNA (1 µg) was individually transfected into HEK293T cells using the TransIT^®^ mRNA transfection kit (MIR 2250, Mirus, Madison, WI, USA). After 24 h, cell lysates were collected and the protein expression of mRNA synthesized in vitro was verified by Western blot analysis. The antibodies used for the Western blot are listed below: anti-MPXV-A29L rRmAb (Vazyme, RM3387); anti-MPXV-A35R rRmAb (Vazyme, RM3395); mouse Anti-MPXV B6R antibody (pvv13501, AntibodySystem, Paris, French); mouse anti-MPXV M1R antibody (AntibodySystem, SAA0283); anti-MPXV-E8L rRmAb (Vazyme, RM3391); β-actin rabbit mAb (AC026, ABclonal, Wuhan, China); HRP-conjugated goat anti-mouse IgG (Abclonal, AS003); and goat anti-rabbit IgG secondary antibody (Sino Biological, SSA004, Beijing, China).

### 2.6. Preparation and Characterization of Mrna-3012-LNP

The Mrna-1017 and Mrna-1995 were mixed in vitro at a mass ratio of 2:3. The Mrna-1017 and Mrna-1995A were mixed in vitro at a mass ratio of 2:3. The Mrna-1017 and Mrna-1266 were mixed in vitro at a mass ratio of 1:1. The Mrna-1017 and Mrna-1380 were mixed in vitro at a mass ratio of 1:1. The Mrna was solubilized using a 20 Mm citrate buffer (Ph 4), while the cationic lipids (SM102), 1,2-distearoyl-sn-glycero-3-phosphocholine (DSPC), cholesterol, and DMG-PEG 2000 were solubilized in anhydrous ethanol. These components were mixed in a molar ratio of 50:10:38.5:1.5 [38]. Subsequently, the lipids and Mrna were combined to form Mrna-LNP using a microfluidic system (Precision Nanosystems, Vancouver, BC, Canada). The mRNA-LNP solution was then diluted 50-fold with DPBS and concentrated using a 50 kDa PES ultracentrifuge tube. The hydrodynamic diameter, zeta potential, and dispersion index of the mRNA-LNP were measured using a Litesizer 500 (Anton Paar, Graz, Austria). The morphology of the mRNA-LNP was observed with a transmission electron microscope (Hitachi HT7800, Tokyo, Japan). Finally, the encapsulation efficiency of the mRNA-LNP was evaluated using the Quant-iT™ RiboGreen™ RNA Analysis Kit (Thermo Fisher Scientific).

### 2.7. Mouse Vaccination

BALB/c mice were randomized into three groups (*n* = 3 per group or *n* = 5 per group). On day 0, the mice received vaccinations of either 10 µg, 20 µg, or 25 µg (the mass of mRNA) of mRNA-LNP, while DPBS served as a placebo control. Immunization was reinforced on day 14 with the same dose of mRNA-LNP. Serum samples were collected on days 10 and 24 following the initial immunization.

### 2.8. Serum-Neutralizing Antibody Assay

Serum-neutralizing antibodies were evaluated using the rTV-Fluc strain [39,40]. Briefly, 100 μL of DMEM was added to a 96-well plate. After performing a 10-fold dilution of the serum sample, 50 μL of the diluted sample was serially diluted three times in the 96-well plate [32,38,41]. Subsequently, 50 μL of the rTV-Fluc virus solution (4 × 10^3^ TCID_50_/mL) was added to the serum sample wells, mixed thoroughly, and incubated at 37 °C for 1 h. Then, 50,000 Vero cells were added to each well and incubated at 37 °C with 5% CO_2_ for 48 h. Following this, 150 μL of supernatant was discarded, and 100 μL of Bright-Glo™ Luciferase Detection Reagent was added, followed by a 2 min incubation at room temperature away from light. Finally, 150 μL of the assay samples were transferred to a 96-well chemiluminescence detection plate, and the luminescence values (RLU) were quantified using a multifunctional imaging enzyme marker (PerkinElmer EnSight, Waltham, MA, USA). Neutralization inhibition was calculated using the Reed–Muench method [41].

### 2.9. Challenge Experiments and Seroprotection Experiments

BALB/c mice (*n* = 3 for each group) received two intramuscular injections of either 10 µg or 25 µg of mRNA-3012-LNP or DPBS. On day 30 after the first immunization, the rTV-Fluc challenged mice subcutaneously at a dose of 7 × 10^6^ TCID_50_ [32]. Twenty-four hours afterward, in vivo imaging was performed to identify bioluminescent signals. For the serotransfer protection studies, four-week-old nude mice were employed. On day 24 post the initial immunization, serum samples were obtained from mice that had been administered two doses of either 10 µg or 25 µg of mRNA-3012-LNP or DPBS. A total of 20 µL of serum from these immunized mice were mixed with 4 × 10^3^ TCID_50_ of the rTV-Fluc and incubated in vitro at 37 °C for one hour. Subsequently, the mixtures were delivered to mice via both intraperitoneal and intravenous routes. Live animal imaging occurred six hours later to assess the in vitro protective efficacy of the serum [32,42]. Furthermore, to evaluate the in vivo protective effect of the serum in the immunized mice more thoroughly, 50 µL of serum was injected intravenously into nude mice, followed by a subcutaneous injection of 4.9 × 10^6^ TCID_50_ of VACV after an hour. Live animal imaging was carried out to gather bioluminescent signals 24 h after the injection.

### 2.10. Evaluation of MPXV-Specific Antibody Titers

MPXV-specific antibody titers in serum were measured using an enzyme-linked immunosorbent assay (ELISA). Briefly, 100 ng of MPXV antigenic protein was added to each well of a 96-well coated plate. After incubating the coated plates at 4 °C for 16 h, the plates were washed six times with 1× TBST (0.2% Tween-20). Subsequently, a 2% BSA albumin blocking solution was added, and the plates were incubated at 37 °C for 2 h. Following this, the plates were incubated again at 37 °C for an additional 2 h. The coated plates were washed, diluted mouse serum was added, and the plates were incubated at 37 °C for 2 h. After washing the coated plates, horseradish peroxidase (HRP)-coupled goat anti-mouse IgG (AS003, Abclonal, Wuhan, China) was added, followed by incubation at 37 °C for 1 h. The coated plates were washed once more, tetra-methylbenzidine (TMB; TIANGEN, Beijing, China) was added, and the plates were incubated for 20 min at room temperature away from light. The reaction was terminated by the addition of hydrochloric acid solution (2 M; Solarbio, Beijing, China), and the absorbance at 450 nm was measured using an enzyme marker (TECAN, Männedorf, Switzerland). MPXV antigenic protein source: A29L (40891-V08E, Sino Biological, Beijing, China), A35R (40886-V08H, Sino Biological), M1R (40904-V07H, Sino Biological), B6R (40902-V08H, Sino Biological), or E8L (40890-V08B, Sino Biological).

### 2.11. Elispot Assay

The evaluation of cellular immune responses in mice that received either the mRNA-3012-LNP vaccine or a placebo was conducted using an Elispot assay kit (MabTech, Nacka Strand, Sweden), focusing on the detection of IL-2, TNF-α, IFN-γ, IL-4, and IL-6 [38,43]. Briefly, BALB/c mice received two doses of 25 µg each of the bivalent mRNA vaccine candidate or the placebo. Spleens were collected on day 30 following the first immunization, and splenocytes were extracted. Following the manufacturer’s protocol, RPMI 1640 (Gibco) enriched with 10% FBS was introduced to the inclusion plate and incubated for 30 min at ambient temperature. Splenocytes were subsequently dispensed into the wells at a concentration of 3 × 10^5^ cells per well and were stimulated using an MPXV-specific peptide library (GenScript, Piscataway, NJ, USA) that included A29L, A35R, B6R, M1R, and E8L. The plates were subjected to four washes with PBS buffer (Gibco) following a 12 h incubation at 37 °C in an atmosphere of 5% CO_2_. Biotinylated antibodies targeting mouse IL-2, TNF-α, IFN-γ, IL-4, or IL-6 were then applied to the plates and incubated for 2 h at room temperature. After further washing, streptavidin-HRP was introduced, and plates were left for an additional hour at room temperature. Once washing was completed, TMB substrate solution was added, leading to the formation of discernible spots on the plate. The plate was concluded by rinsing with deionized water. Finally, the plates were kept in a dark setting for 48 h before image acquisition using the automated vSpot7 Elispot detector from AID.

### 2.12. Flow Cytometry Assay

The live^+^/CD3^+^/CD4^+^/CD44^+^/CD62L^−^ cells represent CD4^+^ Tem cells, while live^+^/CD3^+^/CD8^+^/CD44^+^/CD62L^−^ cells represent CD8^+^ Tem cells. The MPXV-specific peptide libraries of A29L, A35R, B6R, M1R, and E8L were mixed at a peptide concentration of 0.5 μg/mL for each peptide. One million splenocytes were added to each well of a 96-well plate, followed by the addition of the mixed peptide library. The plate was then placed in an incubator at 37 °C with 5% CO_2_ for 12 h. After this incubation, Brefeldin A (5 μg/mL) was added and the cells were incubated for an additional 4 h. Subsequently, the CD16/CD32 antibody was added, and the cells were incubated at 4 °C for 10 min. The cells were washed using cell staining buffer (Bio-Legend, San Diego, CA, USA) and stained with surface markers CD3 (PerCP/Cyanine5.5), CD4 (FITC), CD8 (PE/Cyanine7), CD44 (PE), and CD62L (APC) according to the manufacturer’s instructions. Following this, the splenocytes were washed again before adding Fixable Viability Dye eFluor™ 780 for staining. Analysis was performed using flow cytometry.

### 2.13. In Vivo Toxicity Evaluation

To assess the in vivo toxicity of the vaccine, we examined the levels of crucial biochemical markers associated with the heart, liver, and kidneys in the serum of mice, along with the concentrations of different cytokines in the blood, 48 h post-inoculation with 25 µg of mRNA-3012-LNP. Furthermore, the primary organ tissues of the mice were collected after this 48 h duration. The collected mouse organ tissues were subsequently stained using hematoxylin and eosin (H&E) for histopharmacological analysis.

## 3. Results

### 3.1. Design, Screening, and Characterization of MPXV Bivalent mRNA Vaccine Candidates

**Figure 1:**
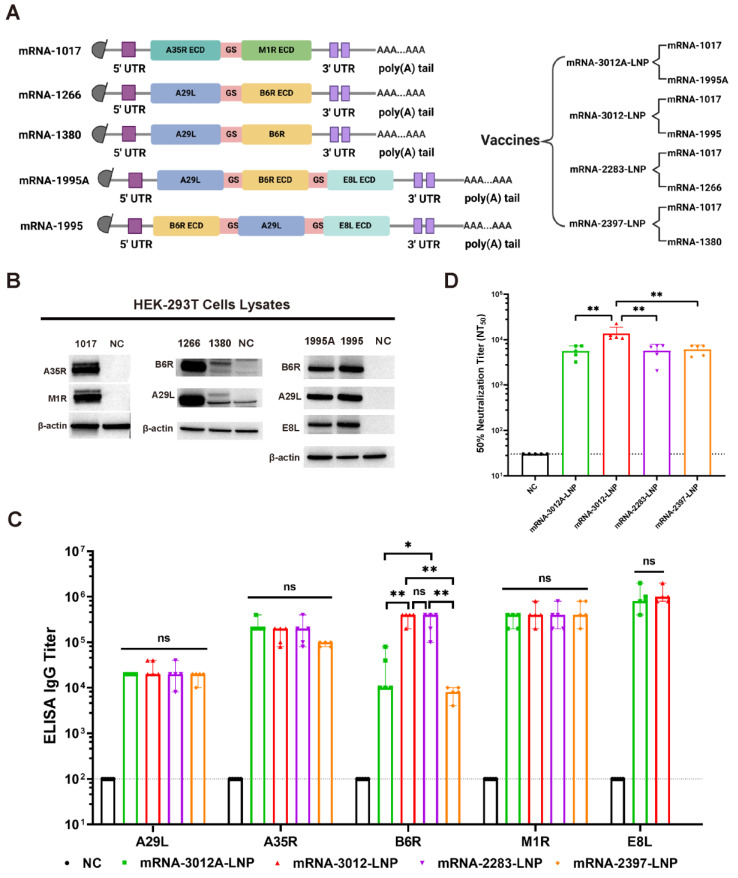
Design, screening, and characterization of MPXV bivalent mRNA vaccine candidates. (**A**) Sequence design of mRNAs and design of a bivalent mRNA vaccine candidate for mpox. ECD, extracellular domain; 5′ UTR, 5′-untranslated region; 3′ UTR, 3′-untranslated region; GS, GS linker. (**B**) The expression of mRNA in cells was analyzed using Western blotting. In vitro synthesized mRNA was transfected into HEK-293T cells, and the cells were lysed 24 h later to collect the lysates, which were subsequently validated by Western blotting. (**C**) The comparison of IgG titers induced by four mRNA vaccines was conducted using ELISA. (**D**) The comparison of neutralizing antibody titers induced by four mRNA vaccines was performed using an rTV-Fluc-based neutralization assay. The data are shown as medians, with dashed lines indicating detection limits. Significance was calculated using the Mann–Whitney test (ns, not significant; * *p* < 0.05 and ** *p* < 0.01).

In this study, the MPXV genome with GenBank ID PP098593.3 was utilized for the design of mpox mRNA vaccine candidates. We designed and synthesized five codon-optimized mRNAs (Figure 1A). Among these, the mRNA-1017 encodes a fusion protein that comprises the extracellular structural domain of A35R and the extracellular structural domain of M1R. Both mRNA-1995 and mRNA-1995A encode fusion proteins that consist of the full-length A29L, B6R extracellular structural domain, and E8L extracellular structural domain; however, they differ in the order of expression of the antigenic proteins. The mRNA-1266 encodes a fusion protein that consists of the full-length A29L and the B6R extracellular domains. The mRNA-1380 encodes a fusion protein that comprises the full-length A29L and the full-length B6R. Utilizing the five mRNAs, we developed a total of four mRNA vaccine candidates. Subsequently, we transfected the five mRNAs into HEK293T cells to verify their expression. Western blot analysis demonstrated that all five mRNAs successfully expressed the corresponding antigenic proteins (Figure 1B). Mpox mRNA vaccine candidates were synthesized using a microfluidic system. We then compared the humoral immunization effects of the four vaccines in BALB/c mice. BALB/c mice were immunized via intramuscular vaccination with two doses of 20 μg of mRNA-2283-LNP or 20 μg of mRNA-2397-LNP or 25 μg of mRNA-3012-LNP or 25 μg of mRNA-3012A-LNP mpox mRNA vaccine candidates, with PBS serving as a placebo control. The two doses of the vaccine were administered 14 days apart. Serum was collected from all mice on day 24 following the initial immunization. The results of the enzyme-linked immunosorbent assay (ELISA) indicated that mice vaccinated with mRNA-3012-LNP or mRNA-2283-LNP produced higher levels of IgG antibodies against B6R compared to the other immunized groups. However, mRNA-3012-LNP also induced high titers of IgG antibodies against E8L compared to mRNA-2283-LNP. In addition, all immunized groups of mice induced high titers of IgG antibodies against A29L, A35R, and M1R (Figure 1C). Furthermore, mice in the mRNA-3012-LNP group exhibited significantly higher titers of neutralizing antibodies against VACV compared to the other immunized groups (Figure 1D). This finding suggests that mRNA-3012-LNP demonstrates a superior immunization effect, leading to its selection for subsequent evaluation. mRNA-3012-LNP had a hydrodynamic diameter of 95.68 ± 0.99 nm and a polydispersity index of 15.1 ± 0.7%. The zeta potential of mRNA-3012-LNP was 15.7 ± 0.3 mV in a physiological pH environment. mRNA-3012-LNP had an encapsulation efficiency of more than 95%, as assessed by Ribogreen fluorescence assay (Figure S1). Transmission electron microscopy (TEM) results showed that mRNA-3012-LNP exhibited a stable solid spherical morphology (Figure S2). In summary, we designed and screened MPXV bivalent mRNA candidate vaccine mRNA-3012-LNP.

### 3.2. Systematic Evaluation of the Effect of mRNA-3012-LNP on Humoral Immunization in Mice In Vivo

**Figure 2:**
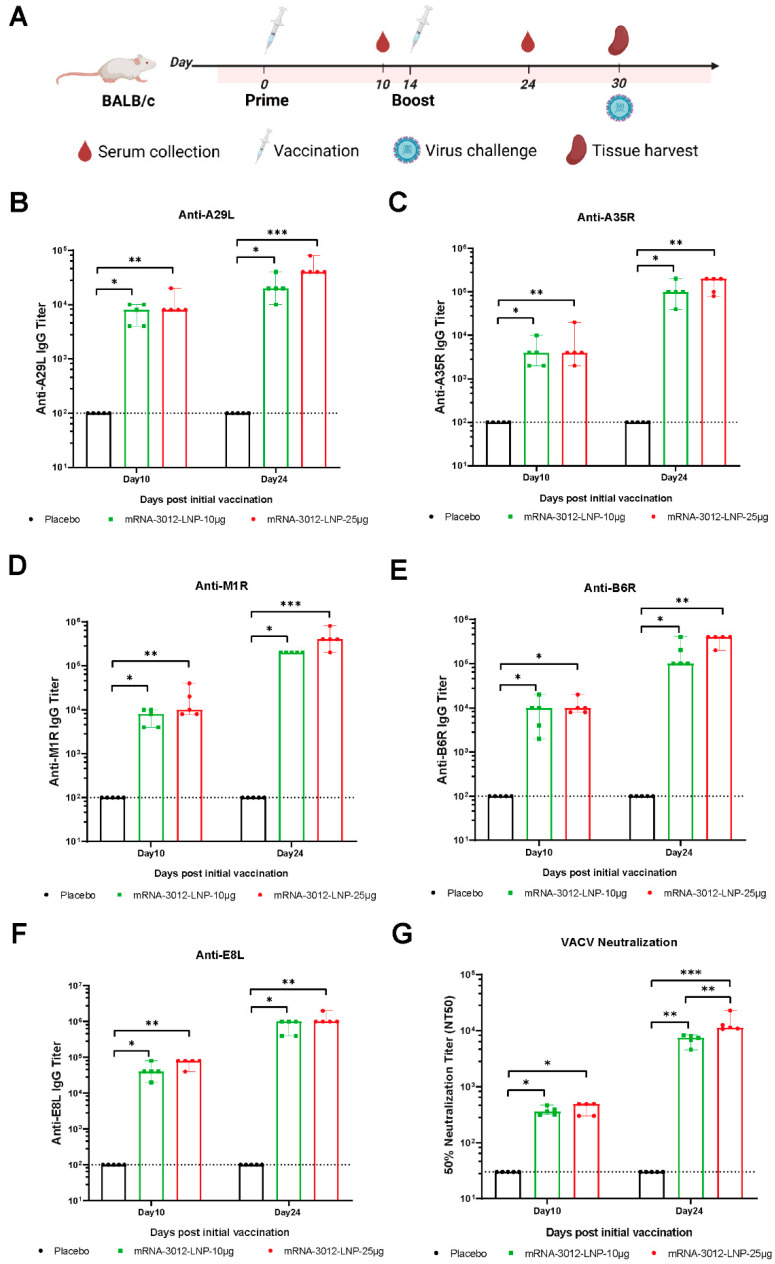
Systematic evaluation of the effect of mRNA-3012-LNP on humoral immunization in mice in vivo. (**A**) The experimental schedule for BALB/c mice encompasses immunization, serum collection, spleen collection, and the timing of viral challenges. (**B**–**F**) Systematic evaluation of mRNA-3012-LNP-induced IgG titers by ELISA. (**G**) Systematic evaluation of RNA-3012-LNP-induced neutralizing antibody titers by rTV-Fluc-based neutralization assay. The data are shown as medians, with dashed lines indicating detection limits. Significant differences were calculated by a Kruskal–Wallis test with Dunn’s multiple comparisons (* *p* < 0.05; ** *p* < 0.01; and *** *p* < 0.001).

The humoral immune response induced by the mpox bivalent mRNA vaccine mRNA-3012-LNP was further systematically evaluated in BALB/c mice. BALB/c mice were injected intramuscularly with two doses of either 10 μg or 25 μg of mRNA-3012-LNP. DPBS served as a placebo control. The two doses were administered 14 days apart. Mouse sera were collected for antibody evaluation on days 10 and 24 following the initial immunization (Figure 2A). After the first immunization, all mice produced IgG antibodies against the five MPXV antigens (A29L, A35R, B6R, M1R, and E8L), with titers exceeding 3 log10 (Figure 2B–F). As determined by ELISA, we observed an increase in the titer of bound antibody against the five MPXV antigens after the second dose of booster immunization compared to the initial immunization (Figure 2B–F). Furthermore, the binding antibody response induced in mice vaccinated with mRNA-3012-LNP exhibited a dose-dependent trend. Additionally, we utilized an rTV-Fluc expressing firefly luciferase to evaluate serum neutralizing antibodies. On day 10 following the initial immunization, neutralizing antibodies against VACV were observed in mice that had been vaccinated with mRNA-3012-LNP. Following the second booster immunization, neutralizing antibodies were significantly increased in all vaccinated mice (Figure 2G). Notably, we observed that the high-dose group elicited approximately twice the level of neutralizing antibodies against VACV compared to the low-dose group (Figure 2G). These results indicated that vaccination with mRNA-3012-LNP elicited a robust humoral immune response in mice, demonstrating a clear dose-dependent trend.

### 3.3. MPXV-Specific Cellular Immune Response in Mice Vaccinated with mRNA-3012-LNP

**Figure 3:**
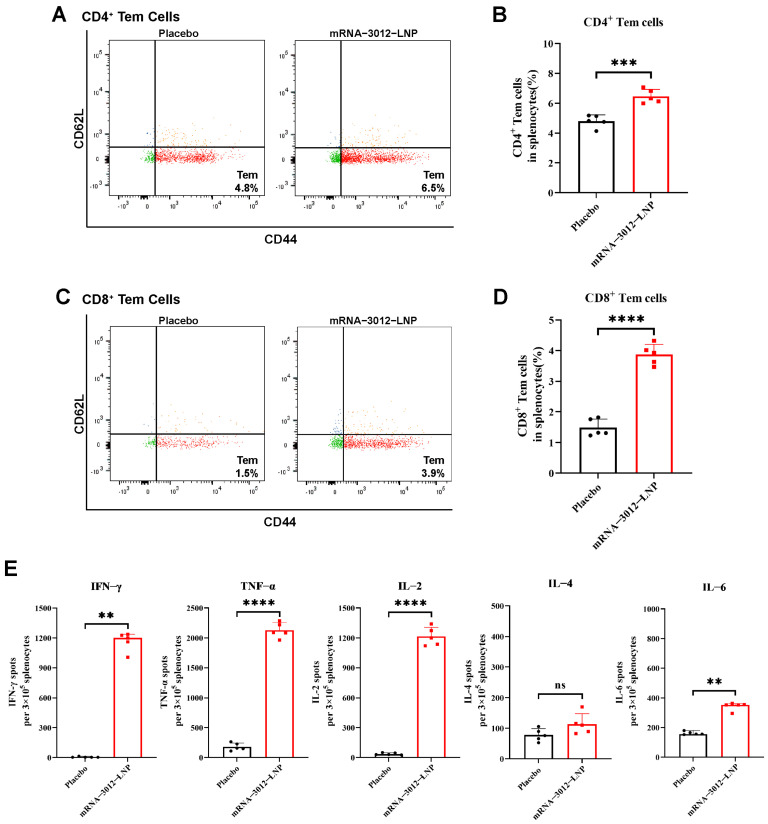
MPXV-specific cellular immune response in mice vaccinated with mRNA-3012-LNP. BALB/c mice (*n* = 5 per group) were immunized intramuscularly with two doses of 25 μg mRNA-3012-LNP, with DPBS serving as the placebo control. On day 30 after the initial immunization, spleens were removed from the mice, and splenocytes were isolated. The evaluation of mRNA-3012-LNP-induced cellular immune responses was conducted following the stimulation of splenocytes using an MPXV-specific peptide library. (**A**–**D**) MPXV-specific CD4^+^ and CD8^+^ Tem cells in mouse splenocytes were assessed by flow cytometry. (**E**) ELISPOT was used to evaluate the level of I cytokines secreted by splenocytes. The data (B, D, IL-2, TNF-α, and IL-4) are presented as mean ± SEM. Significance was calculated using an unpaired *t*-test. The data (IFN-γ and IL-6) are presented as medians. Significance was calculated using the Mann–Whitney test (ns, not significant; ** *p* < 0.001; *** *p* < 0.001 and **** *p* < 0.0001).

To further investigate whether vaccination with a bivalent mRNA candidate for mpox induced a cellular immune response in mice, BALB/c mice were intramuscularly vaccinated with two doses of 25 µg mRNA-3012-LNP, administered two weeks apart [28]. Splenocytes were isolated 30 days after the initial immunization (Figure 2A). Flow cytometry assays demonstrated that the levels of CD4^+^ and CD8^+^ Tem cells in the splenocytes of mRNA-3012-LNP-vaccinated mice were significantly higher than those in the placebo group, following stimulation with MPXV-specific peptide libraries (Figure 3A–D). This finding indicates that our vaccine could provide effective memory protection in mice. Additionally, enzyme-linked immunosorbent spot assays revealed that the levels of interferon-γ (IFN-γ), tumor necrosis factor-α (TNF-α), and interleukin-2 (IL-2) cytokines secreted by splenocytes stimulated with the MPXV peptide library were significantly elevated compared to the placebo group (Figure 3E). In contrast, we did not observe a similar trend in the secretion of interleukin-4 (IL-4) and interleukin-6 (IL-6). This suggests that the MPXV bivalent mRNA vaccine induced Th1-biased T-cell immune responses in mice.

### 3.4. Protection Efficacy of mRNA-3012-LNP Against VACV Challenge in Mice

**Figure 4:**
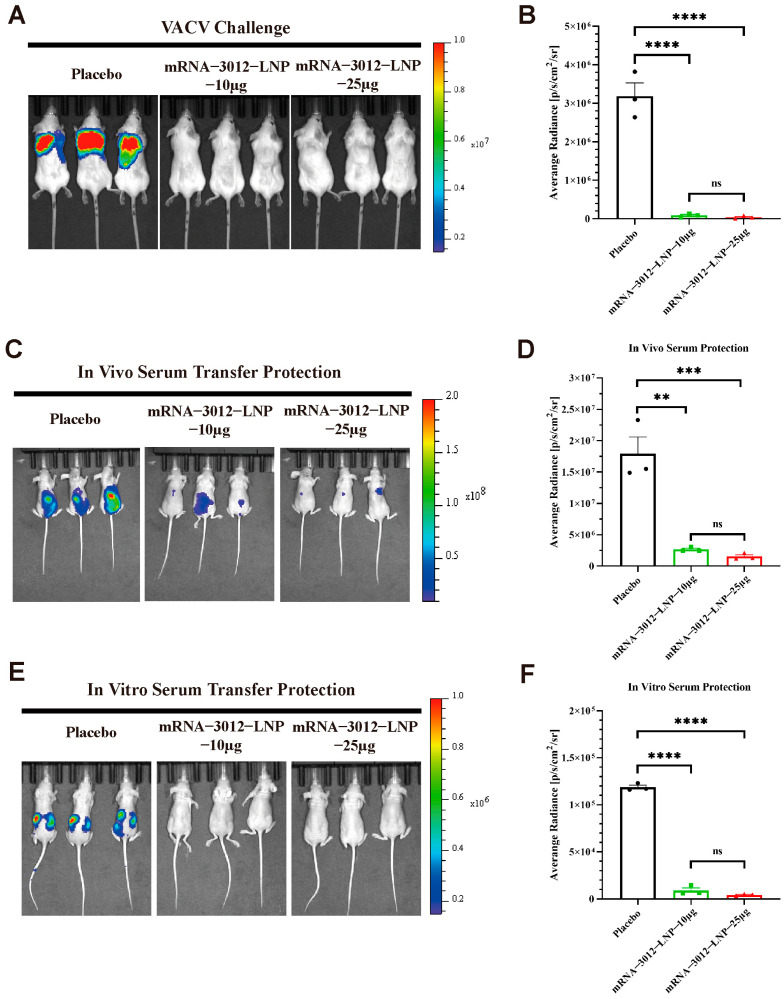
Protection efficacy of mRNA-3012-LNP against VACV challenge in mice. The immunoprotective effects of mRNA-3012-LNP were assessed using a firefly luciferase-based rTV-Fluc experimental animal model. BALB/c mice (*n* = 3) were immunized intramuscularly with two doses of either 10 μg or 25 μg of mRNA-3012-LNP, with the two immunizations administered 14 days apart; DPBS served as a placebo control. Serum was collected from all mice on day 24 after the initial immunization. (**A**,**B**) The protective effect of mRNA-3012-LNP was verified by subcutaneous injection of 7 × 10^6^ TCID_50_ of the rTV-Fluc strain into mice on day 30 post-initial immunization. Bioluminescence measurements were performed on the mice 24 h after the viral challenge. (**C**,**D**) The protective effect of in vitro transfer of mRNA-3012-LNP-immunized mouse serum was evaluated. A total of 20 μL of serum was incubated in vitro with a mixture of 4 × 10^3^ TCID_50_ of the rTV-Fluc virulent strain for 1 h, followed by injection into 4-week-old nude mice (*n* = 3) via tail vein and intraperitoneal injection. Bioluminescence measurements were conducted on the nude mice 24 h later. (**E**,**F**) Evaluation of in vivo transfer protection of mRNA-3012-LNP-immunized mouse serum was assessed by injecting 50 μL of serum into 4-week-old nude mice (*n* = 3) via tail vein. One hour later, 4.9 × 10^6^ TCID_50_ of the rTV-Fluc strain was injected subcutaneously. Bioluminescence measurements were performed on the nude mice 24 h after the viral challenge. Data are shown as mean ± SEM. Significance was calculated by a one-way ANOVA with multiple comparisons (ns, not significant; ** *p* < 0.01; *** *p* < 0.001; and **** *p* < 0.0001).

To further explore whether vaccination with two doses of the mRNA-3012-LNP vaccine provides effective protection for mice challenged by VACV. Previous studies have demonstrated the utility of the firefly luciferase-based VACV Tiantan strain (rTV-Fluc) in assessing vaccine protection efficacy [32,39,42]. In this experiment, three groups of mice were administered two doses of either 10 μg or 25 μg of mRNA-3012-LNP, or a placebo, via intramuscular injection. The doses were given with a 14-day interval between the two vaccinations. A subcutaneous viral challenge was conducted on day 30 following the initial immunization (Figure 2A). Bioluminescent signals were significantly reduced in mice vaccinated with mRNA-3012-LNP 24 h after the subcutaneous challenge with the rTV-Fluc, compared to the placebo group (Figure 4A,B). This finding suggests that our vaccine effectively clears VACV from the mice. Additionally, we assessed whether the serum from mice inoculated with mRNA-3012-LNP exhibited a passive protective effect. To evaluate the in vivo protective effect, sera from either the vaccine or placebo group were injected via the tail vein of nude mice, which were then subjected to a subcutaneous challenge with the rTV-Fluc. Bioluminescent imaging results after 24 h indicated that light signals were significantly reduced in nude mice treated with the immunized serum compared to the placebo group (Figure 4C,D). Subsequently, we verified the in vitro protective effect of the serum. Serum and rTV-Fluc were mixed and incubated in vitro for 1 h before being inoculated into nude mice, with live animal imaging performed 6 h later. The results showed that the bioluminescent signal in nude mice inoculated with the serum from immunized mice was significantly lower than that in the placebo group. This indicates that serum from immunized mice effectively neutralizes VACV in vitro (Figure 4E,F). In conclusion, the mpox bivalent mRNA vaccine candidate demonstrates the ability to provide effective protection by efficiently eliminating VACV that has invaded mice.

### 3.5. Acute and Immunological Toxicity Evaluation of the mRNA-3012-LNP Vaccine in Mice

**Figure 5:**
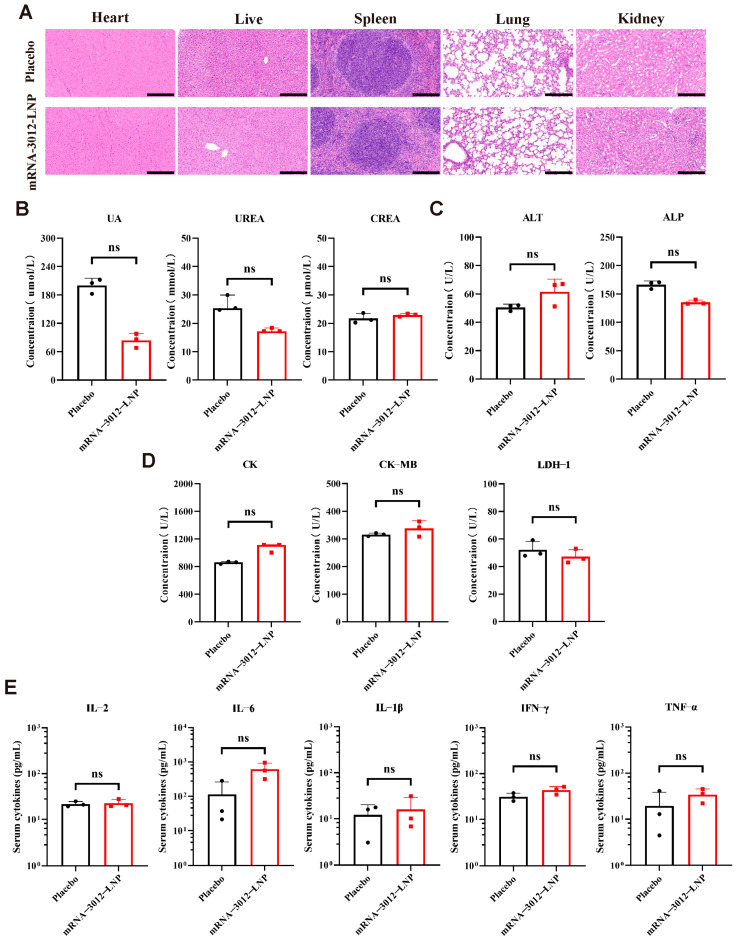
Acute and immunological toxicity evaluation of the mRNA-3012-LNP vaccine in mice. BALB/c mice were immunized intramuscularly with 25 μg of mRNA-3012-LNP, while DPBS served as a placebo control. Serum and major organ tissues were collected 48 h later. (**A**) H&E staining of major organ sections. Scale bar = 200 μm. (**B**–**D**) Detection of biochemical indices in mouse serum, which included kidney parameters (uric acid (UA), urea, and creatinine (CREA)), liver parameters (alanine aminotransferase (ALT) and alkaline phosphatase (ALP)), and heart parameters (creatine kinase (CK), creatine kinase isoenzyme (CK-MB), and lactate dehydrogenase isoenzyme-1 (LDH-1)). (**E**) Assessment of cytokine levels in mouse serum. The data (UA, CREA, ALT, ALP, CK-MB, LDH1, and (**E**)) are presented as mean ± SEM. Significance was calculated using an unpaired *t*-test. The data (IFN-γ and IL-6) are presented as medians. Significance was calculated using the Mann–Whitney test (ns, not significant).

The acute toxicity and immunological toxicity of the MPXV bivalent mRNA vaccine candidate, mRNA-3012-LNP, was further evaluated in vivo. BALB/c mice were inoculated with a single dose of 25 µg of mRNA-3012-LNP or a placebo. Major organ tissues and serum samples were collected 48 h post-inoculation. Histopathology examination through H&E staining of major organ sections revealed no significant pathological damage in the mice vaccinated with mRNA-3012-LNP (Figure 5A). Additionally, the serum levels of key biochemical indices from the kidney (Figure 5B), liver (Figure 5C), and heart (Figure 5D) did not exhibit significant changes in the vaccine group compared to the placebo group. Notably, the absence of a significant inflammatory response is a crucial indicator for assessing the safety of mRNA-LNP formulations [44]. ELISA results indicated that mRNA-3012-LNP did not induce an increase in serum cytokine levels of TNF-α, IFN-γ, IL-2, IL-6, and IL-1β (Figure 5E). In conclusion, these data suggest that mRNA-3012-LNP did not induce strong acute toxicity and immunotoxicity in vivo.

## 4. Discussion

The MPXV outbreak on 14 August 2024, once again constitutes a public health event of international concern [6]. Since the spread of the MPXV epidemic in 2022, there have been over 129,000 confirmed cases and 290 deaths worldwide [7]. In light of the current precarious situation, there is an urgent need to develop novel vaccines for the prevention of mpox infection. The mRNA vaccines were widely utilized during the COVID-19 pandemic due to their efficacy and safety, suggesting that mRNA-based MPXV vaccines hold considerable promise [45,46,47].

Previously, our group designed two tetravalent mRNA vaccine candidates based on mpox virus antigens: mRNA-A-LNP and mRNA-B-LNP [32]. Both vaccines were created by combining multiple single-antigen mRNAs into a single formulation. While this design approach is straightforward and convenient, it increases production costs and complicates quality control analysis. In this study, we designed four mpox multi-antigen tandem bivalent mRNA vaccine candidates and compared their differences in humoral immune responses. The results showed that mRNA-3012-LNP induced a better humoral immune response. Therefore, mRNA-3012-LNP was selected for subsequent evaluation. mRNA-3012-LNP contains two mRNAs: mRNA-1017 and mRNA-1995. mRNA-1017 encodes a fusion protein comprising the A35R extracellular domain and the M1R extracellular domain, while mRNA-1995 encodes a fusion protein comprising the B6R extracellular domain, A29L, and E8L extracellular domains. These antigens are linked using a flexible linker. In comparison to mRNA-A-LNP and mRNA-B-LNP, we utilized the antigenic protein extracellular domain and incorporated the E8L antigenic protein to enhance the efficacy of the MPXV bivalent mRNA vaccine candidate mRNA-3012-LNP, while also aiming to reduce the complexity of the vaccine and simplify the preparation process. Subsequently, we evaluated the immunogenicity and protective efficacy of mRNA-3012-LNP against VACV in a mouse model. As expected, compared with mRNA-A-LNP and mRNA-B-LNP, mice vaccinated with 10 µg or 25 µg of the bivalent mRNA vaccine mRNA-3012-LNP exhibited higher levels of potent neutralizing antibodies against VACV in vivo. This finding suggests that vaccination with either low or high doses of mRNA-3012-LNP can provide effective antibody protection against VACV.

Immune memory serves as the foundation for vaccination [48]. Following vaccination, certain T cells differentiate into effector memory T cells, which recognize pathogens within the body and offer rapid protection by swiftly clearing pathogens upon antigen recognition [49]. Our study revealed a significant increase in CD4^+^ and CD8^+^ Tem cells in the splenocytes of mice vaccinated with mRNA-3012-LNP, aligning with our expectations. In addition, we observed that splenocytes from mice vaccinated with mRNA-3012-LNP, when stimulated with a library of five mpox antigenic peptides, exhibited a significant increase in the secretion of IFN-γ, TNF-α, and IL-2. In contrast, the secretion of IL-4 did not differ significantly from that of the placebo group. These findings suggest that our MPXV bivalent mRNA vaccine elicited Th-1-biased T-cell immune responses against MPXV in mice.

Both MPXV and VACV belong to the genus Orthopoxvirus within the Poxviridae family, and their protein-coding genes are highly conserved. Previous studies have demonstrated that vaccination with an mpox mRNA vaccine can provide effective protection for host animals against VACV infection [33,50]. In the current study, we evaluated the protective efficacy of mRNA-3012-LNP using a VACV challenge model based on firefly luciferase expression [32]. The results indicated a significant reduction in fluorescence intensity in mice inoculated with mRNA-3012-LNP, suggesting that the VACV Tiantan strain (rTV-Fluc) was effectively cleared in the mice. Furthermore, nude mice vaccinated with serum from immunized mice exhibited a strong capacity to resist VACV infection. These findings suggest that our vaccine provides effective protection against VACV. However, our study has certain limitations. It was designed with a focus on the IIb branch of the mpox virus, and the protective effect against the I branch of the mpox virus remains to be investigated. Additionally, our study utilized only VACV and not MPXV for immunoprotection experiments. In future research, the authors plan to address these limitations and achieve a more comprehensive understanding of treatment options for mpox.

## 5. Conclusions

In summary, we report on an MPXV bivalent mRNA vaccine candidate, mRNA-3012-LNP, which induces robust humoral immune responses and MPXV antigen-specific Th-1-biased cellular immune responses in mice, effectively protecting them against VACV. These findings suggest that our vaccine is a promising candidate for the prevention of MPXV infection.

## Data Availability

The data presented in this study are available on request from the corresponding author.

## Supplementary Materials

The following supporting information can be downloaded at https://www.mdpi.com/article/10.3390/vaccines13040374/s1, Figure S1. Physicochemical parameters of mRNA-3012-LNP. Data are presented as mean ± SEM. Figure S2. Transmission electron microscopy image of mRNA-3012-LNP. Scale bar = 200 nm.
