# Supplementary material for: An Mpox Multi-Antigen-Tandem Bivalent mRNA Candidate Vaccine Effectively Protects Mice Against the Vaccinia Virus
Source: Vaccines (Basel). 2025 Mar 31;13(4):374. doi: 10.3390/vaccines13040374 (PMC12031407; doi:10.3390/vaccines13040374)
Supplement: Supplementary file 1 [file vaccines-13-00374-s001.zip › vaccines-3525626-supplementary.pdf]

Physicochemical parameters	Result
**Particle diameter (nm)**	**95.68±0.99**
**Zeta potentials (mV)**	**15.7±0.3**
**Polydispersity index (%)**	**15.1±0.7**
**encapsulation efficiency (%)**	**97.16±0.24**

Figure S1. Physicochemical parameters of mRNA-3012-LNP. Data are presented as mean ± SEM.

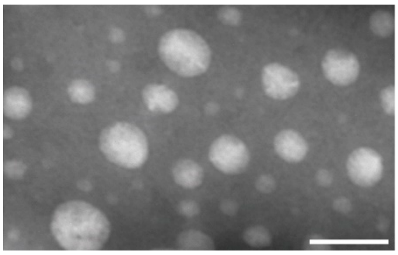

Figure S2. Transmission electron microscopy image of mRNA-3012-LNP. Scale bar = 200 nm.
